# IL-18 and Cutaneous Inflammatory Diseases

**DOI:** 10.3390/ijms161226172

**Published:** 2015-12-09

**Authors:** Ji hyun Lee, Dae Ho Cho, Hyun Jeong Park

**Affiliations:** 1 Department of Dermatology, Seoul St Mary’s Hospital, College of Medicine, the Catholic University of Korea, Seoul 137-701, Korea; ejee@catholic.ac.kr; 2 Department of Life Science, Sookmyung Women’s University, Seoul 140-742, Korea; cdhkor@sookmyung.ac.kr; 3 Department of Dermatology, Yeouido St. Mary’s Hospital, College of Medicine, the Catholic University of Korea, 62 Yeouido-dong, Yeongdeungpo-gu, Seoul 150-713, Korea

**Keywords:** IL-18, psoriasis, atopic dermatitis, urticaria, contact dermatitis, alopecia areata, drug eruption, lupus erythematosus, fibrosis, graft-versus-host disease, skin tumor, skin disease

## Abstract

Interleukin (IL)-18, an IL-1 family cytokine, is a pleiotropic immune regulator. IL-18 plays a strong proinflammatory role by inducing interferon (IFN)-γ. Previous studies have implicated IL-18 in the pathogenesis of various diseases. However, it is not well understood biologic activities of IL-18 in the diverse skin diseases. Here, we have reviewed the expression and function of IL-18 in skin diseases including inflammatory diseases. This article provides an evidence-based understanding of the role of IL-18 in skin diseases and its relationship with disease activities.

## 1. Introduction

Interleukin (IL)-18 (e.g., interferon-γ (IFN-γ)-inducing factor), a member of the IL-1 family of cytokines, is similar, in terms of structure, receptor utilization, and cytokine processing, to IL-1 [1]. IL-18 was purified as an “IFN-γ-inducing factor” from the murine liver [2]. The *IL18* gene is located on chromosome 9 in mice and on chromosome 11 in humans. IL-18 is structurally similar to members of the IL-1 family of cytokines, especially IL-1β and IL-33 [3]. Pro IL-18, an inactive precursor of IL-18, is stored in the intracellular space and is cleaved and processed by caspase-1 into the biologically active cytokine IL-18. After being processed, IL-18 is released into the extracellular milieu. Bellora *et al.* [4], recently revealed that IL-18 is expressed not only as a secreted form but also as a membrane-bound form.

Pro IL-18 has been observed in various cells, including keratinocytes, dendritic cells, macrophages, Kupffer cells, astrocytes, microglia, intestinal epithelial cells, and osteoblasts. The IL-18 receptor (IL-18R) is found on T cells, natural killer (NK) cells, B cells, macrophages, neutrophils, basophils, endothelial cells, smooth muscle cells, chondrocytes, keratinocytes, fibroblasts, melanocytes, and numerous epithelial cells [5,6,7,8,9].

**Figure 1:**
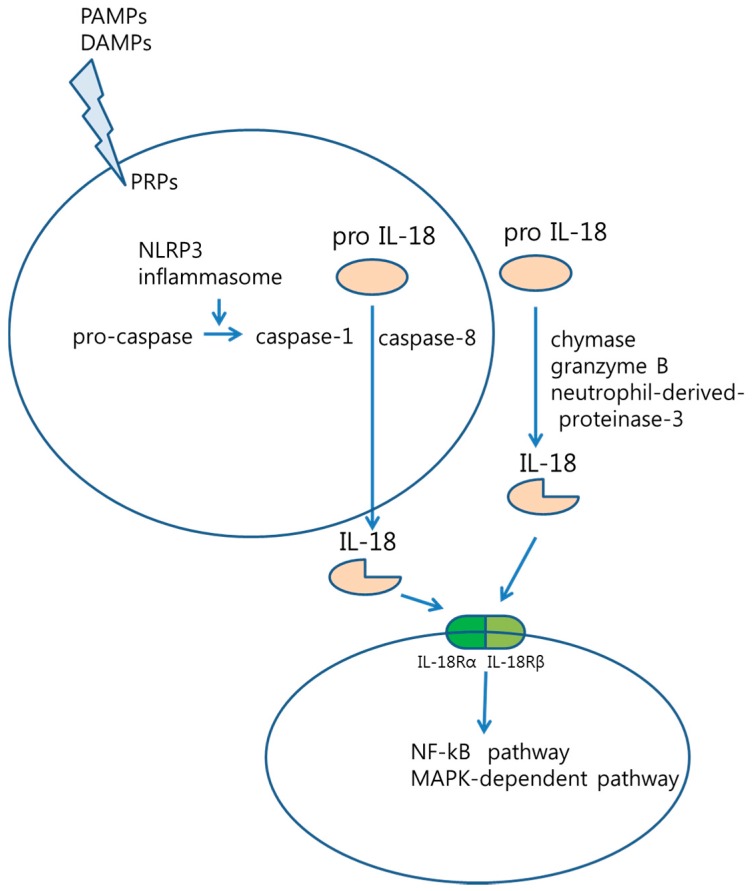
Interleukin (IL)-18 processing stimuli including pathogen-associated molecular patterns (PAMPs) and damage-associated molecular patterns (DAMPs) is detected by pattern-recognition receptors (PRPs), and induce the oligomerization of the nucleotide-binding domain and leucine-rich repeat pyrin domain containing protein-3 (NLRP3) inflammasome. IL-18 is processed and activated by chymase, granzyme B, neutrophil-derived proteinase-3, as well as intracellular caspase-1. In addition, a heterodimer IL-18R is linked with NF-κB pathway. (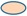: pro IL-18, 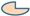: active IL-18, 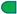: IL-18Rα, 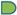: IL-18Rβ).

The inflammasome is a protein complex that mediates innate immune response via the activation of caspase-1 [10]. The activation of inflammasome is initiated by recognition of stimuli, such as pathogen-associated molecular patterns (PAMPs) or danger-associated molecular patterns (DAMPs). Pattern-recognition receptors (PRRs), such as Toll-like receptors (TLRs) or nucleotide oligomerization domain (NOD)-like receptors (NLRs) can detect PAMPs or DAMPs [11]. Oligomerization of the nucleotide-binding domain and leucine-rich repeat pyrin domain containing protein-3 (NLRP3) inflammasome induces the activation of caspase-1 (e.g., IL-1β-converting enzyme, ICE) from pro-caspase-1. Finally, IL-18 is upregulated by the intracellular cysteine protease caspase-1 (Figure 1) [12].

IL-18 is also activated by mast cell chymase, granzyme B, neutrophil-derived proteinase-3 [13], as well as caspases. Caspase-8 activates IL-18 via Fas-associated protein with death domain (FADD).

IL-18R is a heterodimer, composed of α and β chains. Active IL-18 attaches to the α chain of the IL-18R. IL-18Rβ is then recruited to form a signaling complex composed of IL-18, IL-18Rα, and IL-18Rβ. After forming the heterodimer, the intracellular Toll-IL-1 receptor (TIR) domain binds to myeloid differentiation factor 88 (MyD88) and IL-1 receptor-associated kinase (IRAK). In the NF-κB signaling pathway, phosphorylated IRAK binds to tumor necrosis factor (TNF) receptor-associated factor 6 (TRAF6), which degrades inhibitor of κB (IκB). Subsequently, NF-κB is released [12]. Although MyD88-IRAK-TRAF6-NF-κB signaling is the major signaling pathway for IL-18, IL-18 also acts through the phosphorylation of signal transducer and activator of transcription 3 (STAT3) and mitogen-activated protein kinase (MAPK) [14].

Recently, IL-18 binding protein (IL-18BP), a natural IL-18 inhibitor, soluble IL-18 receptors and anti-IL-18 receptor monoclonal antibodies, have been developed to treat inflammatory diseases [15,16]. IL-18BP inhibits IL-18 by competitively binding to IL-18Rs [17]. It inhibits the IFN-γ-inducing capability of IL-18 and may protect against diseases such as contact hypersensitivity.

## 2. IL-18 in Inflammatory and Autoimmune Cutaneous Diseases

IL-18 is generally expressed in a variety of diseases. Its function depends on the surrounding cytokine environment. IL-18 with IL-12 or IL-15 enhances the Th1 response, while IL-18 without IL-12 can stimulate Th2 responses, including allergic inflammation. In the Th1 response, IL-18 induces the expression of the Fas ligand on NK cells and IFN-γ via T cells and NK cells [18]. IL-18 with IL-2 or IL-4 enhances the production of IL-4, IL-13, and immunoglobulin E (IgE) [19]. However, imbalance in IL-18 levels can cause inflammation and various diseases. IL-18 has been implicated in various inflammatory skin diseases, including psoriasis, atopic dermatitis (AD), urticaria, contact dermatitis, alopecia areata (AA), drug allergy, graft-versus-host disease (GVHD), cutaneous lupus erythematosus (CLE), and *etc.* [18,20,21,22,23]. Here, we review the roles of IL-18 in skin diseases so far.

### 2.1. Psoriasis

**Figure 2:**
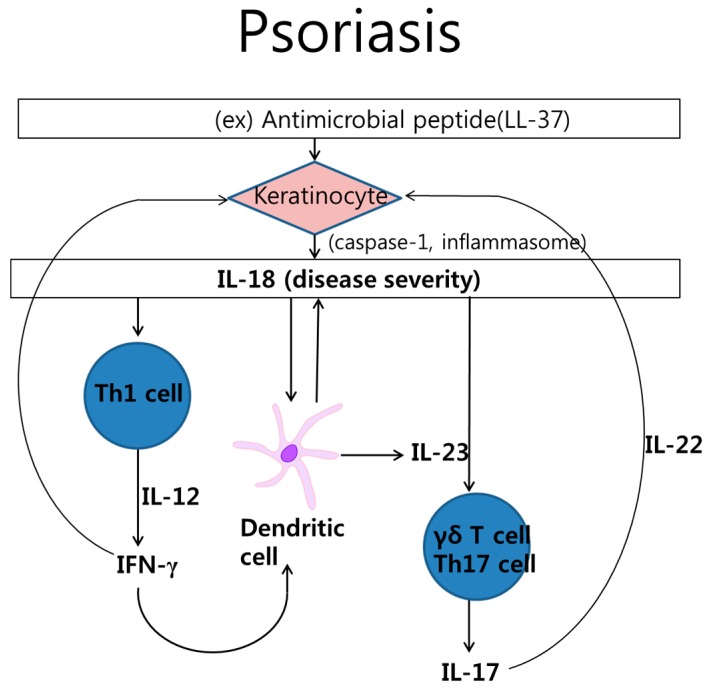
Interleukin (IL)-18 in the pathogenesis of psoriasis. The complex interplay of cytokines and cells is involved pathologic process in psoriasis. Keratinocyte stimulated by cathelicidin LL-37 produces IL-18. IL-18 activated Th1 cell, which produces interferon-γ (IFN-γ). IL-18 also stimulates Th17cell or γδ T cell, which secrete IL-17. In addition, dendritic cell produces IL-18 as well as IL-23.

Psoriasis is a chronic skin disease characterized by abnormal proliferation and unusual differentiation of keratinocytes. It appears well circumscribed, erythematous papules and plaques with silvery thick scales [24]. Psoriasis is associated with a Th1, Th17, and Th22 response [25]. Human keratinocytes constitutively express IL-18 protein. The release of IL-18 from keratinocytes depends on caspase-1 and keratin (Krt) 1, one of the intermediate filament cytoskeleton [26,27]. Previously, we reported that Ultraviolet B(UVB)-induced HaCaT keratinocytes produce IL-18, which is mediated by the production of reactive oxygen intermediates (ROIs) and activation of activator protein (AP)-1 [28]. Antimicrobial peptide, dinitrochlorobenzene, and lipopolysaccharide (LPS) can also stimulate the production of IL-18. Meanwhile, 1,25-dihyroxyvitamin D_3_ decreases IL-18 expression in keratinocytes [29,30]. IL-18 may recruit dendritic cells expressing IL-18R to inflammatory areas under Th1 conditions, as in psoriasis. IL-18 from keratinocytes in synergy with IL-12 plays a role in the Th1 response, primarily by inducing IFN-γ in psoriatic lesions [31]. In addition, besides these classical T helper cells, γδ T cells, and innate lymphoid cells contribute to psoriasis pathogenesis. Furthermore, recent evidence suggests that γδ T cells can produce IL-17 via IL-23 with IL-18 or IL-1β (Figure 2) [32].

Psoriasis patients have increased expression of IL-18 in the skin tissue and blood [33,34]. Caspase-1 is also expressed in psoriatic lesions [35]. Moreover, serum levels of IL-18 positively correlate with the clinical severity score of skin lesions, as is observed with the psoriasis area and severity index (PASI), including chronic plaque-type psoriasis and generalized pustular psoriasis. In particular, IL-18 protein levels in active and progressive psoriatic lesions were higher compared to those in stable and established lesions [36]. IL-18 can be considered a biomarker for the severity of psoriasis.

Interestingly, stress stimulates the hypothalamic-pituitary-adrenal (HPA) axis and sympathetic nervous system. The HPA axis controls corticotrophin-releasing hormone (CRH), α-melanocyte-stimulating hormone (α-MSH), adrenocorticotropin (ACTH), and cortisol. Activation of the HPA axis induces IL-18 and enhances skin inflammation. Acute stress activates the HPA axis and glucocorticoids may induce Th1 responses in the skin, which then aggravate psoriatic lesions. We previously demonstrated that ACTH dose-dependently upregulates IL-18 production via caspase-1, melanocortin receptor, and the p38 and MAPK/ERK pathways in keratinocytes [37]. We also observed that the expression of CRH, a key stress hormone, increased in psoriatic lesions [38]. On the other hand, we showed that CRH decreases the expression of inflammatory cytokines including IL-18 in HaCaT cells via the p38-MAPK pathway [39]. Zhou *et al.* [40], proposed that aberrant CRH system might play a protective role in psoriasis. This phenomenon may reflect that CRH control the negative feedback loop. In a recent study on primary human keratinocytes, IL-18BP was upregulated upon stimulation with IL-27 [41]. In conclusion, these results indicate that studying the changes in the expression of IL-18 will be helpful to further understand of the pathogenesis of psoriasis.

### 2.2. Atopic Dermatitis

**Figure 3:**
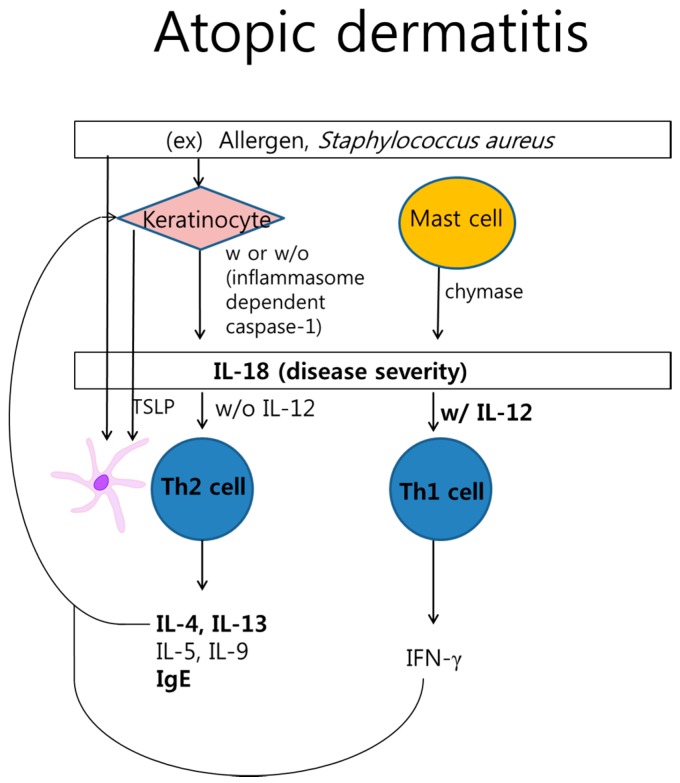
Interleukin (IL)-18 in the pathogenesis of atopic dermatitis. Keratinocyte or mast cell plays IL-18 activation via proteolytic processing. IL-18 can induce Th2 or Th1 cytokines based on IL-12. Dendritic cell activated by allergen or thymic stromal lymphopoietin (TSLP) from keratinocyte interacts Th2 cell.

AD is a chronic skin inflammation with skin barrier dysfunction and intense pruritus. It presents eczematous lesions with dryness and excoriation. The skin lesions of acute AD are papules and vesicles with erythema. Chronic AD becomes thick plaques with lichenificaiton [42,43]. It may be caused by various factors, including genetic predisposition and environmental triggers. AD is thought to initially be driven by a Th2 response involving IL-4 and IL-13, followed by a Th1 response. IL-18 has a pleiotropic effect that stimulates both Th2 and Th1 responses, depending on its cytokine environment (Figure 3). Previous reports revealed that keratinocyte as well as inflammatory dendritic epidermal cell release IL-18 [44]. An elevated expression of serum IL-18 is related to the pathogenesis of AD in children, adults, and AD mouse models [45]. In addition, Konishi *et al.* [46], showed that IL-18 contributes to the spontaneous development of atopic dermatitis-like skin lesions in a transgenic mouse model. IL-18 with IL-12 induces the Th1 response, resulting in the production of IFN-γ. However, Yoshimoto *et al.* [47], showed that IL-18 alone enhances IL-4, IL-5, IL-9, and IL-13 production by basophils, mast cells, and CD4^+^ T cells in helminth-infected IFN-γ mice. Previously, Hoshino *et al.* [48], had demonstrated that IL-18 contributes to CD4^+^ T cell-dependent IgE production. Tsutsui *et al.* [49], suggested that IL-18 directly activates mast cells, which release chymase. Chymase can also cleave pro IL-18 and might accelerate the inflammatory responses in AD lesions. They also suggested that cytotoxic T lymphocytes release perforin and induce the production of granzyme B after recognition of virus-infected keratinocytes, resulting in the activation of pro IL-18. IL-18 stimulates CD1d-dependent natural killer T (NKT) cells, the major source of IL-4. The resulting IL-4 induces immunoglobulin E (IgE) production in B cells. Thus, NKT cells regulate B cell activation via IL-18.

Exposure to allergens such as house dust mites and to lesional infections induces the expression of IL-18. IL-18 contributes to the development of *Staphylococcus aureus*-associated AD in humans as well as in mice [50,51]. In NC/Nga atopic dermatitis mouse model, IL-18 and mast cell had been shown to be important inflammation induced by *Staphylococcus aureus* [51]. Previous studies reported a significant increase of IL-18 by staphylococcal enterotoxin A (SEA) *in vitro* and *in vivo* [52,53]. Monocyte-derived dendritic cells induced by *Malassezia furfur* in peripheral blood stimulate the production of IL-18 in addition to that of TNF-α and IL-1β [54]. Additionally, Zedan *et al.* [55], reported that AD severity (SCORAD score) correlates with the serum IL-18 concentration. Furthermore, chronic stress suppresses the HPA axis and aggravates Th2 immune responses with the lack of immunosuppressive effects in allergic diseases [56]. CRH acts as an anti-inflammatory factor as well as a pro-inflammatory factor, dependent on environmental conditions. Lee *et al.* [57], demonstrated that CRH decreases IL-18 expression in dendritic cells, which express CRH-receptors (CRH-R) in patients with AD. Here, stress may modulate immune responses by influencing the expression of IL-18 in AD. Furthermore, a recent genetic study revealed that single-nucleotide polymorphisms in IL-18, such as IL-18 gene—607C/A (rs1946518) or—137G/C (rs187238), may have a protective effect on AD [58]. Finally, serum IL-18 with crossregulation may be associated with the development and/or manifestations of AD.

### 2.3. Urticaria

Urticaria presents pruritic, well-circumscribed, various sized, erythematous wheals on the whole body with degranulation of mast cells and basophils. It usually fades after a few hours. [59]. Chronic spontaneous urticaria (CSU) is an allergic disease characterized by recurrent itchy wheals that persist for more than six weeks. Intradermal injection of autologous serum can induce CSU in 30%–60% patients. Previously, it was reported that total IL-18 levels correlated with clinical severity scores in the autologous serum skin test (ASST)-positive patients [60]. However, Puxeddu *et al.* [61], demonstrated that significantly increased levels of free IL-18 and IL-18BP were observed in CSU, regardless of the ASST results. Recently, Rasool *et al.* [62], reported that there was no correlation between IL-18 and CSU disease severity. Further studies are needed to define the relationship between the clinical severity of urticaria and IL-18.

Meanwhile, mast cells play a major role in the pathogenesis of various allergic diseases including urticaria. As seen above, chymase, an enzyme in mast cell, can activate pro-IL-18 into mature IL-18 [13]. As a positive feedback, IL-18 and IL-1β activate and modulate mast cells [47]. It has been reported that mast cell in mucosa of mouse is associated withTh2 response by releasing IL-18 [63]. In addition, Schnitzler syndrome is characterized by chronic urticaria and arthralgia, presenting with constitutive activation of the IL-1β/IL-18-producing inflammasome [64]. The precise role of IL-18 of mast cells in urticaria should be studied in terms of innate or adaptive immune responses.

### 2.4. Contact Dermatitis

Contact dermatitis is defined inflammatory cutaneous responses to substance which is composed of irritant contact dermatitis and allergic contact dermatitis [65]. Contact dermatitis presents pruritic, reddish plaques. It is frequently accompanied by oozing blisters or crusts. Irritants or allergens can induce contact dermatitis through the type IV immune response. Irritant and allergic contact dermatitis elicit similar early immune responses through the IL-1 pathway involving IL-18 [66,67]. A hapten stimulates the production of DAMP molecules. DAMP molecule-mediated TLR-2 or -4 induces the expression of proinflammatory cytokines including IL-18 [68]. The inflammasome also reacts to sensitizers such as trinitrochlorobenzene, SDS, and ultraviolet B and triggers the immune system, leading to IL-18 activation and production [69,70]. It was suggested that this pathway involving IL-18 may induce skin sensitization and elicit the development of allergic contact dermatitis [71]. In radiocontrast cells, IL-18R is activated by the MyD88 pathway in the sensitization period of contact dermatitis [72]. Recently, another scenario was suggested: allergens induce oxidative stress and activate the inflammasome and HMGB1-mediated TLR4, resulting in the synthesis of IL-18 [67].

### 2.5. Alopecia Areata

AA is a one type of nonscarring hair loss diseases which usually presented round patches [73]. It is also known as an inflammatory disorder of hair follicle cycling, resulting from the collapse of the anagen-specific immune privilege [74]. Although the specific cause of AA is still unknown, genetic, immunologic, and environmental factors are thought to play a role in the development of AA. IFN-γ is a main factor that induces AA in a murine model [75]. IL-18 is an IFN-γ-inducing factor, as mentioned above. Stress is a well-known triggering and aggravating factor of AA [76]. We observed elevated levels of CRH, ACTH, and α-MSH peptides in the pilosebaceous units of AA [77]. IL-18 is expected to work as a modulator of the HPA axis during stress in AA. Lee *et al.* [78], suggested that the serum level of IL-18 is associated with the prognosis of AA. After taking oral cyclosporine A and methylprednisolone, the mean basal serum level of IL-18 was higher in the responder group than in the poor-responder group. A recent study revealed that single-nucleotide polymorphisms (SNPs) in *IL18* (rs187238 and rs549908) are related to the susceptibility to AA in Koreans [79]. However, large-scale studies of susceptible SNPs are needed to prove the involvement of IL-18 in the development of AA. In summary, IL-18 can be used as an indicator of patient response to AA treatment.

### 2.6. Drug Allergy and Cutaneous Drug Eruption

Cutaneous drug eruption is characterized inflammatory, adverse skin reactions to drugs. It usually presents pruritic, symmetric, morbiliform, or exanthematous rash [80]. The lesions are present elsewhere. Drug eruption can occur through immunologic involvement. We previously reported that imatinib, a specific BCR-ABL tyrosine kinase inhibitor, and antibiotics cause cutaneous drug eruptions with a significant elevation of IL-18 and IL-1β levels [81]. In addition, aspirin-induced acute urticaria accompanies neutrophil activation. IL-18 amplifies acute inflammation and promotes neutrophil recruitment [82]. Kim *et al.* [83], showed that a single nucleotide polymorphism in the promoter region of the *IL18* gene may play a role in the progression of aspirin-induced urticaria. Further research is needed on the role of IL-18 in the pathogenesis of cutaneous drug eruption.

### 2.7. Cutaneous Lupus Erythematosus

Systemic lupus erythematosus (SLE) is an autoimmune, multisystem disease accompanied by the loss of self-tolerance. Cutaneous involvement of lupus erythematosus (LE) is divided LE-specific and LE-non-specific skin manifestations based on histopathologic findings [84]. Acute CLE may present a malar or “butterfly” rash. Discoid LE is the most common form of chronic CLE, which manifests indurated scaly plaques with scarring and pigmentation [85]. In a MRL/MP-*lpr/lpr* (MRL/1) mouse, a lupus murine model, elevated IL-18 was observed in the serum and organs, including the kidney. In this murine model, inhibition of IL-18 led to the delay of disease development and a decrease in disease severity [86,87]. In addition, the expression of IL-18 generally increased in CLE [22]. In subacute CLE, serum IL-18 is higher in antinuclear antibody (ANA)-positive that ANA-negative subjects [88]. TNF-α is a major inflammatory cytokine in lupus and is produced by keratinocytes, fibroblasts, and mast cells in the skin. IL-18 increases the production of TNF-α in these cells, resulting in keratinocyte apoptosis [89]. Thus, it seems that IL-18 regulates the development of systemic and cutaneous lupus erythematosus via TNF-α. IL-18 also induces activation of major histocompatibility (MHC) class II and CXCL10 in inflammatory response [90]. Recent studies also revealed that IL-18, a molecule in the inflammasome pathway, is involved in the dysfunction of endothelial cells, and has deleterious effects on vascular repair in SLE [91]. Neutrophil extracellular traps (NETs) activate IL-18 as a positive loop of inflammasome pathway in SLE [90]. Genetically, single-nucleotide polymorphisms in *IL18* gene are also related to the pathogenesis of lupus. For example, in Asian populations, individuals with rs187238 or rs1946518 are more susceptible to SLE [92,93]. IL-18 may play multiple roles in the development or progression of SLE.

### 2.8. Graft-Versus-Host Disease (GVHD)

GVHD is one of the complications that arise after hematopoietic stem cell transplantation. Acute GVHD presents erythematous macules or patches and may progress to skin necrolysis. Chronic GVHD usually presents lichenoid or sclerodermoid patterns [94]. Acute GVHD is mainly a Th1-response characterized by IFN-γ production. IL-18 enhances the Th1 response by inducing IFN-γ, TNF-α, and GM-CSF, and supports FasL- and perforin-dependent cytotoxic effects [95]. IL-18 is expressed in humans as well as in animal models with acute GVHD. The serum levels of IL-18 correlate with the severity of acute GVHD [96]. We demonstrated, by an immunohistochemical analysis, that enhanced IL-18 expression was found in chronic GVHD (sclerodermoid type) as well as acute GVHD (grade I) [23]. In a mouse model of acute GVHD, administration of anti-IL-18α antibody has demonstrated a protective effect with decreasing apoptosis in the liver and small intestine [97]. Therefore, further investigation about a role of IL-18 in the development and progression of GVHD should be encouraged.

### 2.9. Miscellaneous Diseases

Adult-onset Still’s disease (AOSD) is a systemic inflammatory disease with high spiking fever, leukocytosis, arthralgia, and skin rash. In AOSD, IL-18 plays an important role by inducing Th1 cytokines. IL-18 also related with Th17 response synergistically with IL-23 [98]. Currently AOSD patients are being recruited for clinical trials of recombinant human IL-18BP.

Chronic infantile neurologic, cutaneous, articular (CINCA) syndrome is characterized by persistent skin rash, chronic aseptic meningitis, and extensive polymorphonuclear cell infiltration with upregulated IL-1β and IL-18 [99].

Kampfer *et al.* [100], reported that elevated IL-18 levels were observed during wound healing in mouse models. Sabuncu *et al.* [101], observed increased levels of IL-18 in diabetic foot ulcer.

Do *et al.* [102], showed that expression levels of IL-18 and its receptors are significantly higher in keloids than in normal skin. IL-18 plays a role in keloid pathogenesis through epithelial-mesenchymal interactions. In addition, IL-18 expression was elevated in systemic sclerosis that is a connective tissue disease with abnormal collagen deposition [103]. Pan *et al.* [104], demonstrated that IL-18 acts as a fibrogenic cytokine in hepatic fibrosis or bleomycin-induced lung injury. Meanwhile, we reported that IL-18 downregulates collagen production via the ERK pathway in human dermal fibroblasts [105]. We showed that IL-18 suppresses the expression of type I and III collagen in an IFN-γ-dependent manner. IL-18 also curbs the collagen gene expression when pretreated with the fibrogenic cytokine TGF-β. Therefore, the role of IL-18 in pro- or anti-fibrotic mechanisms under various conditions needs to be further investigated.

## 3. Conclusions

IL-18 is usually activated by caspase-1 in inflammasome. IL-18 plays an important role in the development and/or progression of inflammatory and autoimmune skin diseases. In psoriasis, IL-18 induces Th1 and Th17 responses. On the other hand, in AD, IL-18 promotes Th-1 or Th-2 responses depending on cytokine mileu. IL-18 might be a marker of disease severity in psoriasis, AD, LE, or acute GVHD. However, it is still controversial whether IL-18 levels are related with disease severity in urticaria. Recently, genetic polymorphism has been revealed in a few cutaneous diseases including AD, AA, aspirin-induced urticaria, and LE. IL-18 is involved in an innate immune response (macrophages, NKT cells) as well as in an adaptive immune response. IL-18 mediates signaling network in various dermatologic diseases.

## 4. Future Plans and Expert Commentary

In the present review, we summarized the current knowledge regarding IL-18 in a variety of skin disorders. Taken together, these data suggest that controlling the action of IL-18 or IL-18BP could be a promising method for the treatment of skin diseases.
